# The Impact of Telemedicine in the Diagnosis of Erythema Migrans during the COVID Pandemic: A Comparison with In-Person Diagnosis in the Pre-COVID Era

**DOI:** 10.3390/pathogens11101122

**Published:** 2022-09-29

**Authors:** Giusto Trevisan, Katiuscia Nan, Nicola di Meo, Serena Bonin

**Affiliations:** 1 Department of Medical Sciences, University of Trieste, 34151 Trieste, Italy; 2 ASU GI-Azienda Sanitaria Universitaria Giuliano Isontina, 34128 Trieste, Italy

**Keywords:** erythema migrans, Lyme borreliosis, telemedicine

## Abstract

Background: Erythema migrans (EM) is the hallmark manifestation of the Lyme borreliosis (LB), and therefore its presence and recognition are sufficient to make a diagnosis and to start proper antibiotic treatment to attempt to eradicate the infection. Methods: In this study we compared the clinical data of 439 patients who presented an EM either according to the diagnostic modality through physical assessment or through telemedicine. Conclusions: Our data clearly show that telemedicine for EM diagnosis is useful as it enables prompt administration of appropriate antibiotic therapy, which is critical to avoid complications, especially for neurologic and articular entities. Therefore, telemedicine is a tool that could be adopted for the diagnosis of Lyme disease both by specialized centers but also by general practitioners.

## 1. Introduction

The COVID emergency, through the application of telemedicine tools, has changed the methods for the examination of patients. This has been particularly relevant for skin manifestations diagnosed through teledermatology, online consultations [1,2] and by analyzing the photographs of the patients’ skin lesions [3,4].

In the present study teledermatology was applied to analyze images of erythema migrans lesions and clinical data, including the lesion diameter, provided by patients or their general practitioners. 

Erythema migrans (EM) is the hallmark manifestation of Lyme borreliosis (LB) in its early stage [5], representing a local dissemination of the pathogen, at least in the majority of cases [6]. EM is pathognomonic for LB, and therefore its recognition is sufficient for the diagnosis. This early stage of LB is more often seronegative [7]. LB can affect several organs, including the skin, the nervous system and the joints but also the heart and eyes. Clinical manifestations are linked to specific tissue tropisms specific for *Borrelia burgdorferi* s.l. genospecies [8]: *Borrelia burgdorferi* sensu stricto affects preferably the joints, *Borrelia garinii* and *Borrelia bavarensis* the nervous system, *Borrelia afzelii* and *B. valaisiana* the skin, while among Borreliae of the Lyme group, *Borrelia mayonii* gives highly spirochetemic infections [8]. These features are strictly linked to the clinical differences between LB in Europe and North America, where *Borrelia garinii* and *B. afzelii* are not usually detected, although *Borrelia garinii* has been identified in *Ixodes uriae* in the northwestern Atlantic Ocean [9]. Similarly, in Europe there is no evidence of *B. mayonii* [10].

During the pre-COVID era, EM could be diagnosed and treated by general practitioners [11] or by specialists. The diagnosis of the typical EM is clinical and, when detected, adequate clinical assessment, which may include in some cases serological testing, is required. Nevertheless, antibiotic treatment should be initiated without confirmation by laboratory testing where EM diagnosis is clear [12].

During the COVID pandemic, several public health specialists who worked for ASUGI-Azienda sanitaria universitaria Giuliano Isontina in the region of Friuli Venezia Giulia provided online assistance through teledermatology. This service was extended to patients residing in all Italian regions, allowing a prompt diagnosis of the EM and shortening the time gap between the onset of LB symptoms and antibiotic treatment. Clinical data and images of the skin lesions were provided either by patients or their practitioners. In addition, the availability of teledermatology allowed further monitoring of LB throughout the whole Italian territory [13].

Of note, with regards to possible differential diagnosis of EM, although rarely, certain cases of erythema migrans-like rash have been described in cases of COVID sequalae [14] or after administration of the COVID vaccine [15,16], as well as in patients affected by southern tick-associated rash illness (STARI) [17].

As before the COVID pandemic, telemedicine was usually not applied in Italian hospitals the aim of the present study was at comparing two diagnostic modalities, namely telemedicine versus in-person consultation for the diagnosis of Lyme borreliosis in patients presenting with EM before and during pandemic.

## 2. Results

### 2.1. Demographical Data

During the enrollment period (2014–2022), 439 patients with EM were gathered. Mean age of patients was 41 years (range 1–89 years). Of the entire cohort, 232 were females (mean age 42) and 207 were males (mean age 41). Of these, 203 (106 females and 97 males) were diagnosed before COVID, while 236 (126 females and 110 males) were diagnosed during the COVID pandemic, and 52 children under14 years of age (27 males and 25 females) were included in the entire cohort. Of these, 4 (1 female and 3 males) received a diagnosis before COVID and 48 (24 females and 24 males) during the pandemic. Overall, pediatric patients represented 12% of the entire cohort. Patients were distributed across 19 Italian regions, as summarized in Table 1. The distribution of patients across Italian regions in the two observation periods differed significantly (*p* < 0.001). The center that carried out the study was located in Trieste, one of the major towns of Friuli Venezia Giulia (FVG). The rate of patients who resided far from Friuli Venezia Giulia was significantly different in the two observation periods (*p* < 0.001): before the pandemic, 93 (46%) patients were from FVG and 110 (54%) from other regions, while during pandemic, 19 (8%) came from FVG and 217 (92%) from the other Italian regions. Of the total, 355 (81%) patients lived in northern Italy, which is endemic for LB, and of these 174 (40%) were diagnosed before COVID, while 181 (41%) were diagnosed during the pandemic. 

### 2.2. Tick Bite and Anatomical Sites

Of the entire cohort, 370 patients (84%) recalled a tick bite, while 69 did not. The distribution of these patients during the observation period resulted in significant differences, as shown in Table 2, with a higher number of patients that did not recall the tick bite during the pandemic period (*p* = 0.04).

Patients presented with EM rashes in different anatomical sites as shown in Table 3. Overall, the anatomical distribution of the skin lesions before the COVID pandemic was similar to that during the COVID period (*p* = 0.6). In addition, there were no significant differences between genders (*p* = 0.4) in the anatomical sites of the EM, neither in the pre-COVID period (*p* = 0.7) nor during the pandemic (*p* = 0.3).

**Figure 1:**
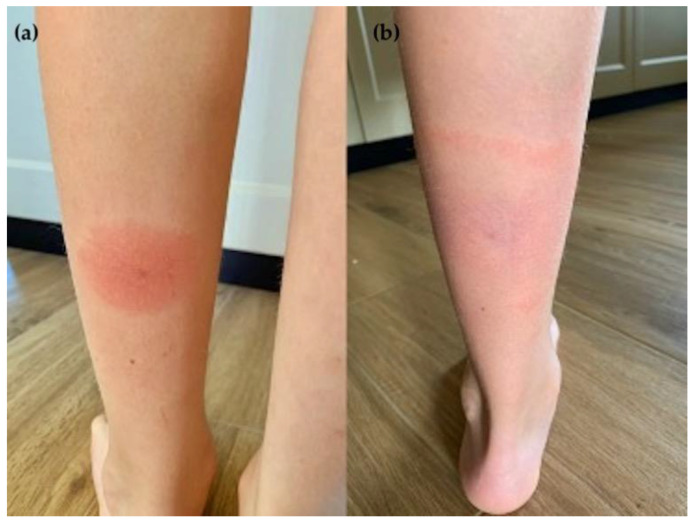
Evolution of the erythema migrans (**a**) at early appearance and (**b**) after 8 days.

Of the EM rashes observed on the upper limbs, 2.4% were on the hands and, among the EM in the lower limbs, 2.9% were on the feet, and 2.9% of the EM rashes were located on the trunk around the breast and mammary areola. Of the rashes affecting the pelvis, 4 were observed on the genitals. Overall, among adults the EM was located more frequently in the lower regions of the body (54%) as shown in Figure 1 rather than in the upper part (46%), both before (51% lower limbs vs. other 49%) and during COVID pandemic (57% lower limbs vs. other 43%) (*p* = 0.2).

For the pediatric patients subgroup, no further subdivision was made besides the observation period due to the limited number of patients diagnosed before the COVID pandemic. The anatomical distribution of the EM rashes observed is reported in Table 4. 

Similar to the adult patients, among the pediatric cases there were no significant differences between males and females (*p* = 1.0) regarding the anatomical distribution of the EM, neither before (*p* = 0.5) nor during the COVID pandemic (*p* = 0.9). Although the number of pediatric patients before the pandemic was lower than during (4 vs. 48 cases), the distribution of the anatomical sites of EM did not vary significantly in the two observation periods (*p* = 0.9), as reported in Table 4.

### 2.3. Symptoms

Erythema migrans was the only symptom in 144/439 cases (33%), while in 292 (67%), other associated clinical manifestations were recorded, as reported in Appendix A, Table A1. In detail, 39 patients (19%) diagnosed before the COVID pandemic presented only with the EM rash, while during the COVID pandemic the patients who only presented the EM rash were 105 (45%). Consequently, before COVID 164 patients (81%) also had symptoms associated with EM, while during the COVID pandemic (*p* < 0.001) the number of patients who presented other symptoms was 130 (55%). The distribution of associated symptoms, including fever, headache, and lymphadenopathy, was similar in terms of observation periods (*p* = 0.5) and in terms of gender (*p* = 0.6), both before (*p* = 0.3) and during the pandemic (*p* = 0.9) (see Table 5). However, analyzing in detail the organs and the systems involved in LB in our cohort, the neurological (headache excluded) (66 patients, 15%) and the articular manifestations (117 patients, 26%) were significantly more common in the patients examined in the pre COVID period in comparison to those diagnosed during the pandemic (7% and 16%) (*p* < 0.001). During COVID, on the other hand, skin manifestations were more frequent (42 patients, 18%) than in the pre-COVID period (36 patients, 8%) (see Table 5 for details). Organs and systems involvement was similar with respect to gender (*p* = 0.4), both before (*p* = 0.7) and during the pandemic (*p* = 0.3). 

### 2.4. Serological Tests and Treatment

Serological tests to confirm LB diagnosis were carried out in 93% of patients before COVID and in 72% during the pandemic (*p* < 0.001). Despite the difference in the number of patients tested, the rate of positive serological tests was similar in the two groups (*p* = 0.1), as reported in Table 6. 

The time lapse between the EM onset and initiation of treatment varied significantly depending on the observation periods (*p* < 0.001) being longer before the COVID pandemic (Table 7). During the pandemic, 58% of patients started treatment within 1 month from the onset of the EM, while in the pre-COVID period only 14% did so. It is worth noticing that before COVID, 97 patients (48%) started the antibiotic treatment after 3 months from the onset of the rash, while during COVID, the number dropped to 22 patients.

Three cases of Jarisch–Herxheimer reaction were observed at the beginning of treatment. The patients were female (4 years, 42 years, and 52 years), and two of them were treated before COVID with amoxicillin, while the third was treated during the pandemic with doxycycline. All women experienced fever and multiple annular erythema. The 52-year-old woman treated with doxycycline also experienced diplopia. 

Among the female patients, three were pregnant. All of them were treated with amoxicillin and their children were born healthy. Details on those patients are reported in Appendix A, Table A2.

429 out 439 patients (203 males and 226 females) were treated with antibiotic therapy. Some patients had been prescribed the initial antibiotic cycle by other medical facilities. Ten patients, although they had received a diagnosis at other centers, were not prescribed antibiotic treatment, and therefore those patients are included in the second cycle treatment as detailed in Table A4 of the Appendix B, where information regarding the first cycle is also reported. Some patients, before turning to our healthcare facility were prescribed other treatments, such as cortisone administered systemically, methotrexate, or ciprofloxacin, as reported in detail in Appendix B. In the pre-COVID group, treatment with first- or second-line antibiotics was carried out in 197/203 (97%) patients, of which 94 were men and 103 were women. During the COVID pandemic, 232/236 (98%) received antibiotic therapy, of which 109 were men and 12 were women.

In total, 150 out 439 patients (34%) were subjected to a second antibiotic cycle, with 97 out 203 (48%) before COVID and 53 out 236 (22%) during the pandemic (*p* < 0.001). Details regarding treatments are shown in Appendix B.

## 3. Discussion

This study explores the use of teledermatology in comparison to in-person consultation for the diagnosis of erythema migrans, i.e., Lyme borreliosis. The group including patients who received in person consultations refers to the period between 2014 and 2019, before the COVID pandemic, while the group made up of patients diagnosed through telemedicine is related to the pandemic period. Before the COVID pandemic, telemedicine was usually not applied in most Italian hospitals. In-person consultations of course mostly involved the patients’ regions of residence, in particular Friuli Venezia Giulia (46%), while during the COVID pandemic a significantly higher rate of patients who resided far from the consultation center were able to receive a virtual consultation and be included in the study thanks to telemedicine. Overall, in both observation periods, most consultations involved patients from northern Italy, which is endemic for LB. However, during COVID pandemic 23% of patients came from Italian regions that are not considered endemic. Of those patients most recalled the acquisition of the tick bite in the region where they lived, highlighting the presence of LB throughout the entire country, although incidence rates fluctuate. To support our findings, *B. burgdorferi* sensu stricto and *B. afzelii* were isolated from *Ixodes ricinus* ticks in southern Italy [18].

Compared to the pre-pandemic period, during the pandemic a significantly higher number of patients did not recall the tick bite. This could more likely be due to the lockdown and the other measures taken to control the spread of COVID rather than to the way in which the consultation took place or by LB itself. Social isolation during lockdown has been reported to impact cognitive abilities, including memory [19], possibly explaining our findings.

As expected, the anatomical distributions of the EM rashes were similar in the two observation periods, with a prevalence of rashes affecting the lower limbs in adults in comparison to the upper parts of the body. In pediatric patients, although lower limbs were confirmed as the most frequent site (17 out 52, 33%), 29% (15 out 52) of EM rashes were observed on the head and neck, while only 8% (35 out 439) of the adult cohort had the EM rash in these areas, in agreement with Backman and colleagues [20]. Pediatric consultations increased by 10 times during the COVID pandemic. This could be mostly due to the fact that before COVID, most pediatric patients were diagnosed by their pediatricians rather than by specialized dermatologists. Our results are in line with other authors highlighting that during COVID lockdown telemedicine proved to be an effective way to provide long distance advice regarding skin lesions in children [21]. Although telemedicine and image analysis have already been applied in pediatrics [22], in agreement with us, it has been proven useful especially during the COVID pandemic [23].

Interestingly, the rate of patients presenting with an EM and associated manifestations was significantly higher before the COVID pandemic, although general symptoms, such as headache, fever, and lymphadenopathy [24,25] were observed in similar numbers in the two observation groups. Nevertheless, the organs and systems involved in LB in the two groups differed significantly with the joints and muscular system being more affected in the group that underwent the in-person consultations, and skin lesions (other than EM) were more prevalent in the teledermatology group. This result can be related to the fact that during COVID pandemic mostly patients with skin manifestations could benefit from the remote assistance. Neurological symptoms, with the exception of headaches, because of the non-specificity, resulted as more prevalent before COVID (66 out 164-40% vs. 17 out 130-13%). The main reason could be the shorter time lapse between the onset of the EM and the beginning of the antibiotic treatment in the teledermatology group [26,27]. It was already reported that teledermatology during the pandemic showed an advantage of being time effective for patients [28]. 

EM is pathognomonic for LB, and therefore there is no need for further analysis in order to make a diagnosis, including serological tests, which can be negative and therefore are not usually recommended [29]. The number of serological tests performed before COVID was significantly higher as a possible consequence of restrictions during the COVID pandemic. Nevertheless, the rate of serological positivity was similar in both groups. Antibody assays were normally suggested to patients with late EM or with other associated manifestations [30], where positive serology is expected. Teledermatology resulted in a significantly shorter time lapse between the onset of symptoms and beginning of therapy with 58% (138 out 236) of patients who started their treatment within 1 month, whereas only 14% (29 out 203) of the patients in the “in-person consultation” group were able to start treatment in the same lapse of time. During the COVID pandemic, 85% (201 out 236) of patients started their therapy within 2 months, while the percentage dropped to 35% (72 out 203) for the previous period, when therapy was started in most cases between months 3 and 12 months (60% vs. 11%). Therapy was seldom initiated after 1 year in both groups. As already reported, the therapy delay has important clinical implications in patients with LB, as it is strongly associated with treatment failure [31]. The application of teledermatology during the COVID pandemic in this study resulted therefore in an improvement of the diagnosis and treatment process. Nevertheless, in some cases during the pandemic, muscular and joint symptoms were misdiagnosed for COVID symptoms, causing delayed diagnosis and, consequently, worsening LB clinical manifestations [32]. 

The antibiotics prescribed for the first round of treatment were mostly amoxicillin and doxycycline, with similar rates in the two observation periods, while ceftriaxone and penicillin G, which are administered intravenously, were mainly prescribed to the “before COVID” group since this treatment protocol is mostly given to patients with late LB [33]. During the first antibiotic cycle, three cases of Jarisch–Herxheimer reaction were observed. All three cases which occurred at the start of antibiotic therapy were of moderate severity and were characterized by fever and annular erythema, as already shown for Lyme group Borreliae [34]. Symptoms regressed with continuation of therapy within 48–72 h. 

During the COVID pandemic, three pregnant women with EM were also under our care. They were treated with amoxicillin within 1–2 months from the EM onset, and, as previously shown [35], the treatment prevented any complication to the babies who were born healthy.

Of our cohort, patients previously subjected to inadequate treatments (10) as well as patients who remained symptomatic after the first antibiotic cycle were submitted to a second antibiotic cycle [36]. The number of these patients was significantly higher in the “in-person consultation group”, maybe as a consequence of the diagnosis and treatment delay.

During the COVID pandemic, telemedicine has been accepted as an effective tool to provide virtual visits and remote care [37,38,39]. Our data clearly show that the use of tele-medicine for EM diagnosis is helpful as it allows shorter delays in antibiotic therapy and consequently fewer neurological and articular complications. Therefore, telemedicine could be a tool that could be adopted in the diagnosis of Lyme disease both by Lyme diseases specialized centers but also by general practitioners. It is indeed worth mentioning that the EM does not always form the characteristic bull’s eye pattern, but several forms have been observed, including the erisipeloid or purpuric variants [40], the bullous form [41], as well as other atypical variants [42]. 

We acknowledge as a limitation in this study that other signs and symptoms suggesting possible tick-borne coinfections [43], such as with *Anaplasma phagocytophilum* [44], were not investigated. Nevertheless, our results show that the application of telemedicine had some benefits from patients, such as time effectiveness in the diagnosis and treatment preventing the progression of LB in some cases. 

## 4. Materials and Methods

This retrospective study comprises patients with proven Lyme borreliosis by the diagnosis of erythema migrans (EM). Patients presented either local infection or disseminated disease. Recruitment, inclusion, and follow-up of participants occur both online and at the clinical expert center for Lyme borreliosis in the University hospital of Trieste. The main inclusion criterion was a diagnosis of EM provided by an expert dermatologist (G.T., N.d.M., K.N.). Additional data refer to other symptoms at diagnosis, such as serological as well as molecular analyses supporting the diagnosis (if present) and the therapy.

For each patient the following data were recorded: age at diagnosis, gender, place of residence, the memory of a tick bite, the anatomical site of EM, additional symptoms, serological tests [45], previous therapies, and the antibiotic therapy given by our specialists. Serology was based on two-tier testing, including the Chemoluminescence Immunoassay (ChLIA catalog number LI 2132-10010, Euroimmun Italia Srl, Padova, Italy) and the Western Blot/Immunoblot assay (Line Blot catalog number DN 2131, Euroimmun Italia Srl, Padova, Italy) as a confirmatory test in case of positive or borderline ChLIA test. Positive, negative, as well as equivocal results were defined according to the manufacturer’s instructions. 

Patients were split into two groups according to the date of diagnosis and to the application of telemedicine. Group 1 included patients diagnosed in person from 01/01/2014 to 31/12/2019 while Group 2 included patients diagnosed by telemedicine from 01/01/2020 to 31/03/2022.

Only patients who gave their informed consent to use their clinical data and images for scientific publications were enrolled in this study.

Data distribution was investigated by Shapiro–Wilk normality test. For continuous variables, comparisons between groups were performed using parametric (Student’s *t* test) or non-parametric tests (the Kruskal–Wallis test) according to the variables’ distribution. The Pearson’s chi-square test was used for categorical variables. A *p*-value < 0.05 was considered statistically significant. Statistical analyses were performed using Stata/SE 16.1 software (StataCorp, College Station, TX, USA).

## 5. Patients

In this study were enrolled patients with erythema migrans diagnosed from 01/01/2014 to 31/03/2022 at the dermatology unit of the University Hospital of Trieste.

## Figures and Tables

**Table 1:** Patient distribution across Italian regions before and during the COVID pandemic. Regions are listed in alphabetical order. Northern Italian regions are in bold.

Italian Regions	Before COVID	During COVID	Total
Abruzzo	0	1	1
Basilicata	0	0	0
Calabria	2	4	6
Campania	2	4	6
**Emilia Romagna**	**19**	**28**	**47**
**Friuli-Venezia Giulia**	**93**	**19**	**112**
Lazio	4	7	11
**Liguria**	**5**	**11**	**16**
**Lombardy**	**16**	**57**	**73**
Marche	3	2	5
Molise	1	3	4
**Piemonte**	**8**	**32**	**40**
Puglia	4	3	7
Sardinia	1	3	4
Sicily	4	5	9
Tuscany	8	22	30
**Trentino-Alto Adige**	**5**	**5**	**10**
Umbria	0	1	1
**Valle d’** **Aosta**	**3**	**4**	**7**
**Veneto**	**25**	**25**	**50**

**Table 2:** Recollection of tick bite according to observation period.

Tick Bite	Before COVID	During COVID	Total
Yes	179/203 (88%)	191/236 (81%)	370/439 (84%)
Unknown	24/203 (12%)	45/236 (19%)	69/439 (16%)

**Table 3:** Anatomical distribution of erythema migrans. M, male; F, female.

Anatomical Site	Total			Before COVID			During COVID		
	All (%)	M (%)	F (%)	All (%)	M (%)	F (%)	All (%)	M (%)	F (%)
Head and neck	35 (8)	15 (7)	20 (9)	16 (8)	6 (6)	10 (9)	19 (8)	9 (8)	10 (8)
Higher limbs	63 (14)	33 (16)	30 (13)	32 (16)	17 (18)	15 (14)	31 (13)	16 (15)	15 (12)
Trunk	80 (18)	43 (21)	37 (16)	39 (19)	21 (22)	18 (17)	41 (17)	22 (20)	19 (15)
Pelvic groin	23 (5)	14 (7)	9 (4)	13 (6)	7 (7)	6 (6)	10 (4)	7 (6)	3 (2)
Lower limbs	238 (54)	102 (49)	136 (58)	103 (51)	46 (47)	57 (54)	135 (57)	56 (51)	79 (63)

**Table 4:** Anatomical distribution of erythema migrans in pediatric patients.

Anatomical Site	Total			Before COVID	During COVID
	All	Male	Female	All	All
Head and neck	15 (29%)	7 (26%)	8 (32%)	1 (25%)	14 (29%)
Higher limbs	3 (6%)	2 (7%)	1 (4%)	0 (0%)	3 (6%)
Trunk	15 (29%)	8 (30%)	7 (28%)	2 (50%)	13 (27%)
Pelvic groin	2 (4%)	1 (4%)	1 (4%)	0 (0%)	2 (4%)
Lower limbs	17 (32%)	9 (33%)	8 (32%)	1 (25%)	16 (33%)

**Table 5:** Summary of the clinical symptoms in the cohort of patients before and during COVID pandemic.

Erythema Migrans	Total			Before COVID			During COVID		
	All (%)	M (%)	F (%)	All (%)	M (%)	F (%)	All (%)	M (%)	F (%)
W/o other manifestations	144 (33)	69 (33)	75 (32)	39 (19)	19 (20)	20 (19)	105 (45)	50 (45)	55 (44)
With associated symptoms	294 (67)	138 (67)	156 (68)	164 (81)	78 (80)	86 (81)	130 (55)	60 (55)	70 (56)
**Associated Symptoms ^1^**									
Fever	80 (18)	38 (19)	42 (18)	46 (23)	22 (23)	44 (41)	33 (14)	19 (17)	14 (11)
Headache	86 (20)	43 (21)	43 (19)	42 (21)	17 (18)	25 (24)	41 (17)	22 (20)	19 (15)
Lymphadenopathy	22 (5)	13 (6)	9 (4)	10 (5)	6 (6)	4 (4)	12 (5)	7 (6)	5 (4)
**Organs and Systems Involved ^1^**									
Joints	156 (36)	73 (35)	83 (36)	117 (58)	55 (57)	62 (59)	39 (17)	18 (16)	21 (17)
Skin	78 (18)	32 (16)	46 (20)	36 (18)	16 (17)	20 (19)	42 (18)	16 (15)	26 (21)
Muscle	169 (38)	91 (44)	78 (32)	106 (52)	59 (61)	47 (49)	59 (25)	32 (29)	27 (21)
Neurological	139 (32)	64 (31)	75 (32)	88 (43)	41 (42)	47 (44)	51 (22)	23 (21)	28 (22)
Neurological w/o headache	83 (19)	34 (16)	49 (21)	66 (33)	30 (31)	36 (34)	17 (7)	4 (4)	13 (10)
Heart	29 (7)	11 (5)	18 (8)	16 (8)	6 (6)	10 (9)	13 (6)	5 (5)	8 (6)
Eyes	38 (9)	19 (9)	19 (8)	20 (10)	9 (9)	11 (10)	18 (8)	10 (9)	8 (6)

^1^ Percentages are calculated with respect of the total number of patients, before and during COVID pandemic and with respect to gender.

**Table 6:** Serological test data.

Serology	Total		Before COVID		During COVID	
	Patients	%	Patients	%	Patients	%
No	79/439	18%	14/203	7%	65/236	28%
Yes	360/439	82%	189/203	93%	171/236	72%
**Serological Results**						
Positive	300/360	83%	152/189	80%	148/171	87%
Negative	60/360	17%	37/189	20%	23/171	13%

**Table 7:** Time lapse from diagnosis to first antibiotic treatment (mo = months). In brackets are reported the percentage values.

First Treatment	<1 mo	>1 mo <2 mo	>2 mo <3 mo	>3 mo <6 mo	>6 mo <12 mo	>12 mo
All	167 (38)	106 (24)	47 (11)	61 (14)	40 (9)	18 (4)
M	75 (36)	56 (27)	21 (10)	27 (13)	19 (9)	9 (4)
F	92 (40)	50 (22)	26 (11)	34 (15)	21	9 (4)
**Before COVID**						
All	29 (14)	43 (21)	34 (17)	51 (25)	36 (18)	10 (5)
M	12 (12)	24 (25)	15 (16)	23 (24)	18 (19)	5 (5)
F	17 (16)	19 (18)	19 (18)	28 (26)	18 (17)	5 (5)
**During COVID**						
All	138 (58)	63 (27)	13 (6)	10 (4)	4 (2)	8 (3)
M	63 (57)	32 (29)	6 (5)	4 (4)	1 (1)	4 (4)
F	75 (59)	31 (25)	7 (6)	6 (5)	3 (2)	4 (3)

## Data Availability

Data available on request to trevisan@units.it.

## Appendix A

**Table A1:** Clinical symptoms of the enrolled patients.

**Skin Manifestations (Different from Erythema Migrans)**			
	Total	Before COVID	During COVID
Total	78	36	42
Multiple Annular Erythema	60	26	34
Borrelia Lymphocytoma	10	6	4
Acrodermatitis chronica atrophicans	1	0	1
Urticaria	7	4	3
**Neurological Manifestations (Headache Excluded)**			
	Total	Before COVID	During COVID
Total	83	66	17
Cranial nerves	21	14	7
Garin Bujadoux-Bannwarth	18	15	3
Meningo-encephalitis	3	2	1
Paresthesia	28	21	7
Anxiety/Depression	10	7	3
Cognitive Disorders	29	21	8
**Joints Manifestations**			
	Total	Before COVID	During COVID
Total	156	117	39
Migratory arthralgia	127	103	24
Temporo-mandibular joint	32	28	4
Arthritis	30	15 (3 with synovial fluid)	15
Sacroiliitis	2	2	0
**Eyes Manifestations**			
	Total	Before COVID	During COVID
Total	38	20	18
Conjunctivitis	26	12	14
Photophobia	8	5	3
Diplopia	5	3	2
Optical neuritis	3	2	1
**Cardiac Manifestations**			
	Total	Before COVID	During COVID
Total	29	16	13
Arrhythmia	26	14	12
Pericarditis	3	2	1

**Table A2:** Clinical details of pregnant patients.

ID	Age	Group	Associated Symptoms	Anatomical Site EM	Time Lapse EM-Therapy	Serological Results	Antibiotic Therapy	Mother	NewBorn
1	30	During COVID	Headache	Trunk (Back left)	<1 mo	IgM+IgG−	Amoxicillin 1 g 3×/die - 14 days	Healed (10 days)	Healthy
2	32	During COVID	None	Lower limbs right	>1 < 2 mo	IgM+IgG+	Amoxicillin 1 g 3×/die - 14 days	Healed (6 days)	Healthy
3	29	During COVID	None	Higher limbs right	<1 mo	IgM+IgG+	Amoxicillin 1 g 3×/die - 14 days	Healed (10 days)	Healthy

## Appendix B

In the following tables, data on antibiotic therapy are reported.

**Table A3:** Details of the first antibiotic cycle.

Antibiotics (I or II Choice)	Amoxicillin/ POM-Penicillin/Cefuroxime	Doxycycline/Minocycline	Azithromycin/ Clarithromycin	Ceftriaxone iv/Penicillin G iv
**Total**				
All	199	171	30	29
M	92	83	10	18
F	107	88	20	11
**Before COVID ^1^**				
All	79+2+3	67+11	7+4	23+1
M	35+1+2	37+2	1+2	14+0
F	44+1+1	30+9	6+2	9+1
**During COVID ^2^**				
All	111+1+3	90+3	13+6	5
M	52+1+1	42+2	5+2	4
F	59+0+2	48+1	8+4	1

^1^ Before COVID pandemic 203 patients were submitted to therapy: 197 to antibiotic therapy (94 males and 103 females), 2 patients were treated with quinolone drugs, 1 patient with methotrexate, 2 patients (1male and 1 female) with betamethasone, and 1 patient (female) with prednisone. ^2^ During COVID pandemic 236 patients were subjected to therapy: 232 to antibiotic therapy (109 males and 123 females), 1 patient (male) was treated with cefpodoxime, 1 patient with deltacortene (1 female), and 2 patients (2 females) with betamethasone.

In all, 150 patients were subjected to a second antibiotic cycle (65 males and 85 females). Of these, 97 were included in the group before the COVID pandemic (40 males and 57 females), while 53 were included during the pandemic (25 males and 28 females).

**Table A4:** Details on the second antibiotic cycle.

Antibiotics	Amoxicillin/Cefuroxime	Doxycycline/Minocycline	Azithromycin	Ceftriaxone iv/Penicillin G iv
**Total**				
All	44+4	35+5	5	51+6
M	21+2	13+2	1	25+1
F	23+2	22+3	4	26+5
**Before COVID**				
	22+4	18+5	2	40+6
M	9+2	5+2	1	20+1
F	13+2	13+3	1	20+5
**During COVID**				
	22+0	17+0	3	11+0
M	12	8	0	5
F	10	9	3	6
